# ERRATUM of Issue 209 JNMA 2018

**DOI:** 10.31729/jnma.3835

**Published:** 2018-10-31

The online version of the “Clinico-radiological Observations in Meconium Aspiration Syndrome”(Lama S, Mahato S, Chaudhary N, Agrawal N, Pathak S, Kurmi O, Bhatia B, Agarwal K. Clinico-radiological Observations in Meconium Aspiration Syndrome. J Nepal Med Assoc. 2018 Feb;56 (209):510–5) has been updated according to the letter to the editor and respective authors' reply published in JNMA 213 Issue.

